# Emergency Cholecystectomy for Gangrenous Acute Cholecystitis with Profound Thrombocytopenia: Immune Thrombocytopenia Diagnosed Postoperatively

**DOI:** 10.70352/scrj.cr.26-0596

**Published:** 2026-09-04

**Authors:** Teppei Tokumaru, Motoyasu Tabuchi, Sunao Uemura, Shuta Tamura, Hiroki Iriyoshi, Toshi Imai, Mototsune Kakizaki, Sayaka Osawa, Takehiro Okabayashi

**Affiliations:** 1 Emergency and Critical Care Center, Kochi Health Sciences Center, Kochi, Kochi, Japan; 2 Department of Gastroenterological Surgery, Kochi Health Sciences Center, Kochi, Kochi, Japan; 3 Department of Hematology and Blood Transfusion, Kochi Health Sciences Center, Kochi, Kochi, Japan; 4 Department of Pathology, Kochi Health Sciences Center, Kochi, Kochi, Japan; 5 Department of Anesthesiology, Kochi Health Sciences Center, Kochi, Kochi, Japan

**Keywords:** gangrenous acute cholecystitis, immune thrombocytopenia, platelet transfusion, cholecystectomy, profound thrombocytopenia, source control

## Abstract

**INTRODUCTION:**

The optimal source-control strategy for gangrenous acute cholecystitis in patients with profound thrombocytopenia remains unclear. Moreover, infection-associated and immune thrombocytopenia may be difficult to distinguish during the acute phase.

**CASE PRESENTATION:**

A 76-year-old man with hypertension, diabetes mellitus, and a history of open distal gastrectomy for gastric cancer presented with gangrenous acute cholecystitis and profound thrombocytopenia (2000/μL). Infection-associated consumptive thrombocytopenia was initially considered, and emergency open cholecystectomy was performed following platelet transfusion. Thrombocytopenia persisted despite source control, and the response to platelet transfusion was suboptimal. The overall clinical course, exclusion of alternative causes, and preserved trilineage hematopoiesis on bone marrow examination supported a clinical diagnosis of immune thrombocytopenia. Treatment with dexamethasone, *Helicobacter pylori* eradication therapy, prednisolone, and rituximab resulted in platelet recovery. The patient was discharged on POD 16.

**CONCLUSIONS:**

This case highlights the necessity of balancing urgent source control against substantial bleeding risk in gangrenous acute cholecystitis with profound thrombocytopenia. Persistent thrombocytopenia following surgical source control, particularly with a suboptimal response to platelet transfusion, should prompt reassessment of the underlying etiology, including immune thrombocytopenia.

## INTRODUCTION

Severe acute cholecystitis requires timely source control, such as cholecystectomy or gallbladder drainage.^1,2)^ Although current transfusion guidelines recommend prophylactic platelet transfusion before major surgery in patients with severe thrombocytopenia, the optimal source-control strategy for acute cholecystitis in this setting remains unclear.^1–3)^ In patients with severe infections, thrombocytopenia may initially be attributed to infection-associated consumption.^4,5)^ However, persistent thrombocytopenia despite adequate source control, particularly when accompanied by a suboptimal response to platelet transfusion, should prompt reassessment of the underlying etiology, including immune thrombocytopenia.^4–6)^ Herein, we present a case of gangrenous acute cholecystitis with profound thrombocytopenia requiring emergency open cholecystectomy after platelet transfusion, in which immune thrombocytopenia was diagnosed postoperatively.

## CASE PRESENTATION

A 76-year-old man presented with general fatigue and poor oral intake that had begun the day prior to admission. His medical history included hypertension, diabetes mellitus, and open distal gastrectomy with Roux-en-Y reconstruction for gastric cancer 4 years earlier, followed by adjuvant chemotherapy. His regular medications had remained unchanged for several years. He had no history of cytopenia and no evidence of recurrent gastric cancer.

On admission, he was alert and hemodynamically stable, with a blood pressure of 111/56 mmHg, heart rate of 80 beats/min, respiratory rate of 22 breaths/min, body temperature 36.7°C, and oxygen saturation of 96%. Physical examination revealed localized tenderness in the right upper quadrant.

Laboratory tests showed a white blood cell count of 8030/μL, hemoglobin level of 14.4 g/dL, and platelet count of 2000/μL. The immature platelet fraction was elevated at 22.3%. The prothrombin time–international normalized ratio was 1.10, activated partial thromboplastin time was 30.6 s, fibrinogen concentration was 890 mg/dL, and fibrin/fibrinogen degradation product concentration was 25.5 μg/mL. The C-reactive protein level was 32.96 mg/dL. The Japanese Association for Acute Medicine disseminated intravascular coagulation score was 6.^7)^ Peripheral blood smear examination showed neither schistocytes nor platelet clumping.

**Fig. 1:**
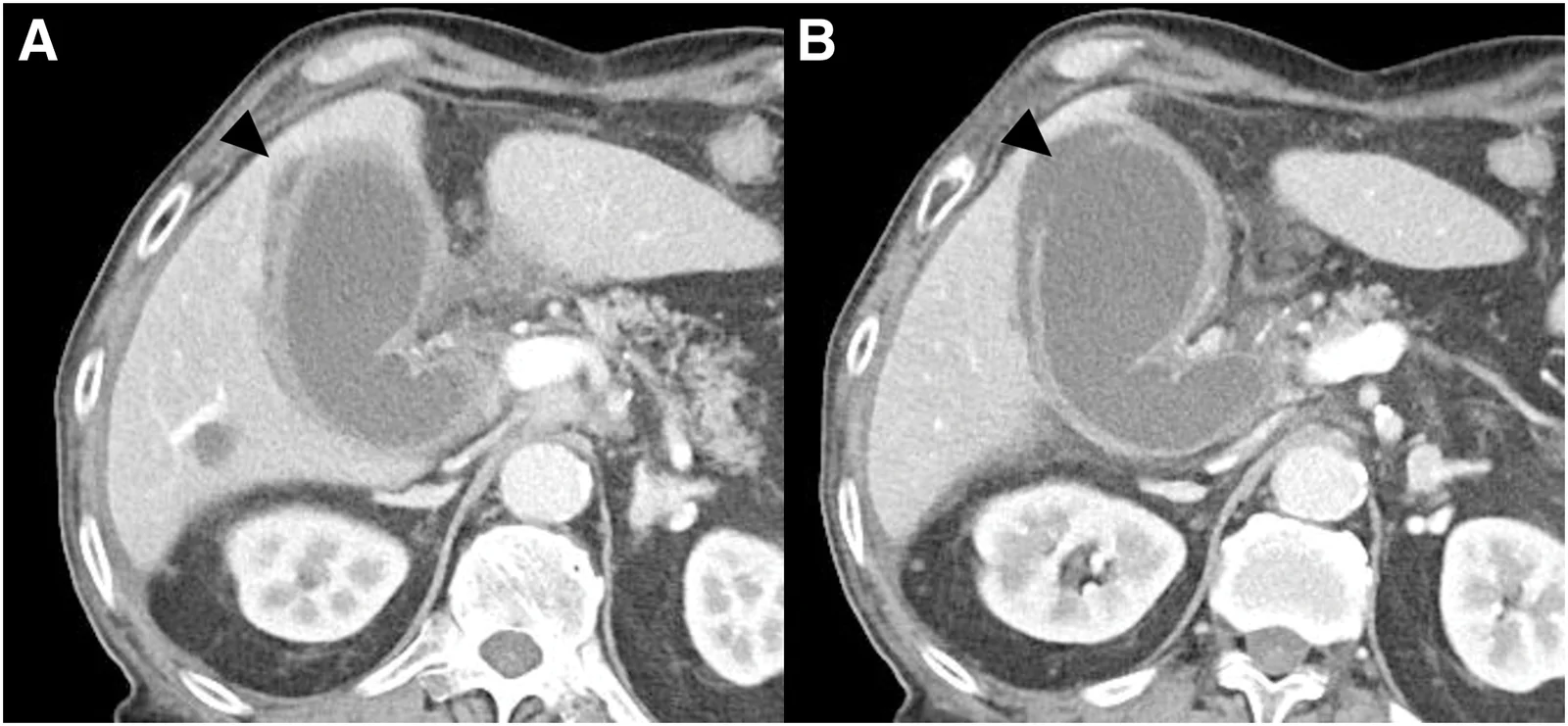
Contrast-enhanced CT findings. (**A**) Pericholecystic fluid collection (arrowhead) with irregular thickening of the gallbladder wall. (**B**) Focal non-enhancement of the gallbladder wall (arrowhead). The findings were suggestive of gangrenous acute cholecystitis.

Contrast-enhanced CT revealed irregular thickening of the gallbladder wall, a partially non-enhancing area, and pericholecystic fluid collection, consistent with gangrenous acute cholecystitis accompanied by a pericholecystic abscess (**Fig. 1**). Neither splenomegaly nor lymphadenopathy was observed. According to the Tokyo Guidelines 2018, the condition was classified as Grade III acute cholecystitis,^8)^ and the American Society of Anesthesiologists physical status (ASA-PS) classification was III. Owing to the severe local infection, infection-associated thrombocytopenia, including disseminated intravascular coagulation, was initially considered, and urgent source control was prioritized.

Percutaneous transhepatic gallbladder drainage and cholecystectomy were considered. We were concerned that drainage alone might not remove the necrotic gallbladder and the related infectious focus; thus, cholecystectomy was selected as a tailored approach to control the source. Substantial intraoperative bleeding was anticipated, and intra-abdominal adhesions related to the previous upper abdominal surgery were expected; therefore, we selected a planned open surgery to facilitate adhesiolysis, direct compression, and rapid hemostasis.

One dose of platelet concentrate, equivalent to approximately 2.0 × 10^11^ platelets, was transfused immediately before skin incision. Since surgery was initiated immediately following transfusion, a post-transfusion platelet count was not obtained prior to incision. Emergency open cholecystectomy was initiated 4 h post-admission.

**Fig. 2:**
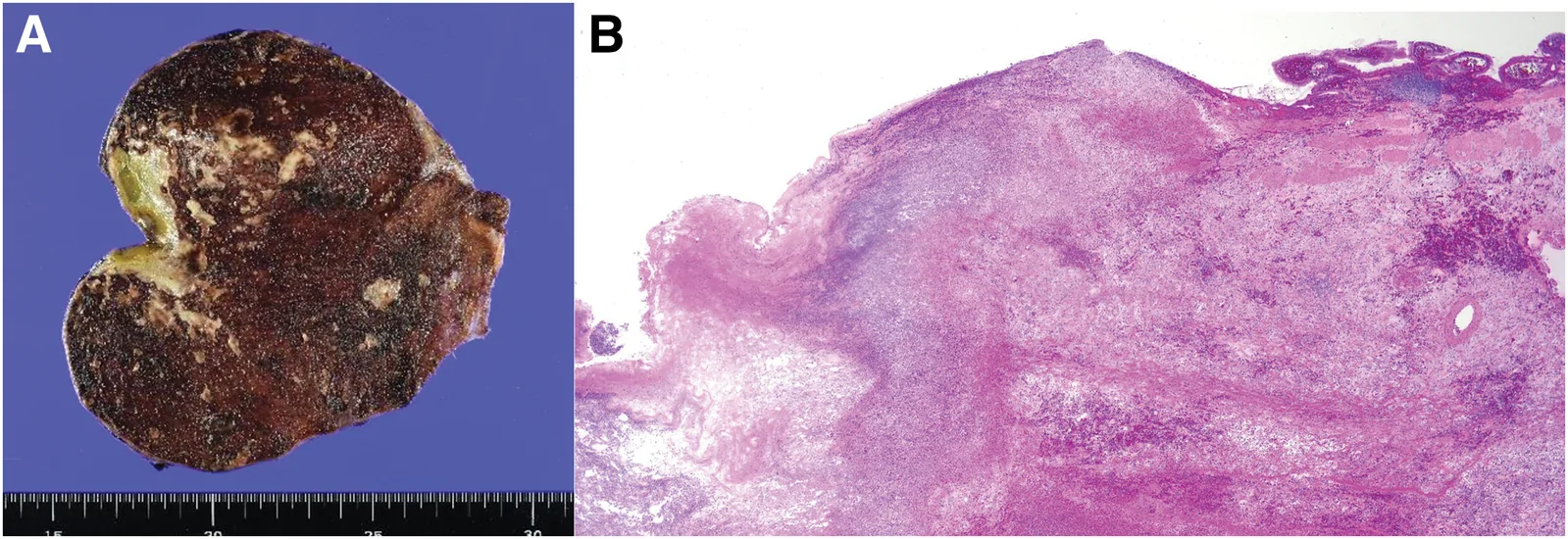
Macroscopic and histopathological findings. (**A**) Macroscopic appearance of the formalin-fixed resected gallbladder showing marked wall thickening with extensive hemorrhagic and necrotic changes. (**B**) Histopathological examination showing extensive transmural necrosis and dense inflammatory cell infiltration, confirming gangrenous acute cholecystitis (hematoxylin and eosin staining, original magnification ×20).

Intraoperatively, the gallbladder was necrotic and accompanied by pericholecystic abscess formation. Cholecystectomy was completed despite a marked bleeding tendency. The bleeding was predominantly diffuse oozing from the dissected surfaces, particularly the gallbladder bed, and hemostasis was achieved via compression and soft coagulation. The operative time was 149 min, and the estimated blood loss was 1570 mL. Two units of red blood cells and 4 units of fresh frozen plasma were administered intraoperatively, without additional platelet transfusion. Histopathological examination confirmed gangrenous acute cholecystitis (**Fig. 2**).

On POD 2, the platelet count remained low at 6000/μL, despite surgical source control. After transfusion of 1 dose of platelet concentrate, the platelet count increased to 12000/μL at 1 h.

The 1-h corrected count increment was 5100, suggesting a suboptimal response to platelet transfusion.^6)^ Persistent severe thrombocytopenia, poor post-transfusion platelet recovery, an elevated immature platelet fraction, the absence of schistocytes and splenomegaly, and preserved trilineage hematopoiesis without dysplasia or blast proliferation on bone marrow examination collectively supported a clinical diagnosis of immune thrombocytopenia. Intravenous dexamethasone at 16.5 mg/day was initiated on POD 2 and continued for 4 days.^9)^

**Fig. 3:**
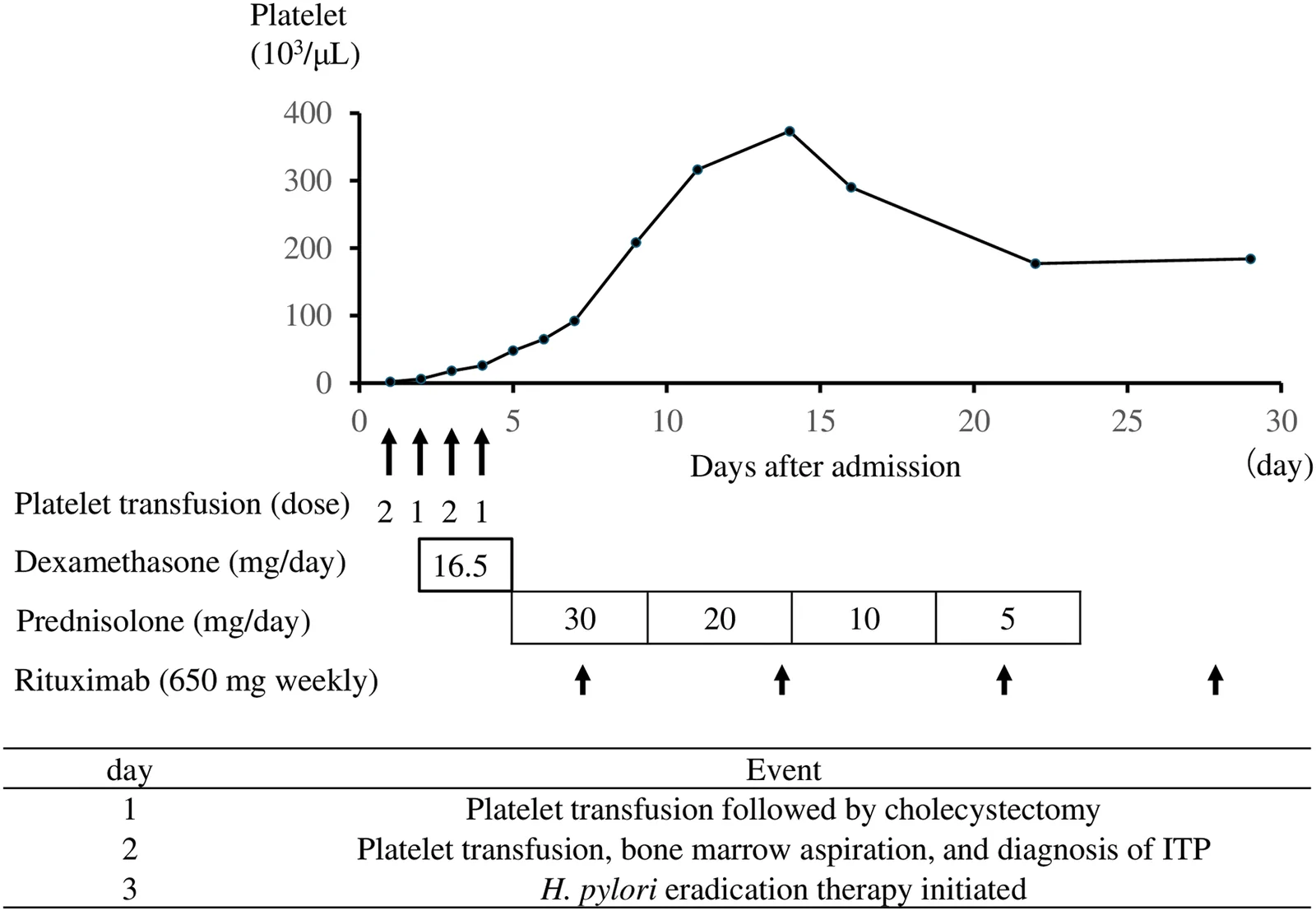
Clinical timeline. Time course of platelet count and treatments following admission. Persistent thrombocytopenia after source control and a suboptimal response to platelet transfusion prompted reassessment on POD 2. Bone marrow aspiration helped to exclude alternative hematologic causes, and the overall findings supported a clinical diagnosis of immune thrombocytopenia. Platelet recovery was observed after subsequent treatment.

As serum anti-*Helicobacter pylori* antibody testing was positive, eradication therapy was initiated on POD 3. Prednisolone was initiated after completion of dexamethasone therapy. Rituximab was initiated as second-line therapy on POD 7 at 375 mg/m^2^ (650 mg/body) and administered once weekly for 4 doses.^9)^ The platelet count subsequently increased, and no further platelet transfusions were required after POD 4 (**Fig. 3**). No major postoperative or treatment-related complications occurred, and the patient was discharged on POD 16. At the 4-month follow-up, he remained clinically stable without recurrence of severe thrombocytopenia.

## DISCUSSION

We report a case of gangrenous acute cholecystitis with profound thrombocytopenia (2000/μL) in which immune thrombocytopenia was diagnosed after emergency open cholecystectomy. This case has 2 important clinical implications. First, in gangrenous acute cholecystitis with profound thrombocytopenia, the need for urgent source control must be balanced against the risk of substantial perioperative bleeding, and the treatment strategy should be individualized. Second, persistent thrombocytopenia after source control, particularly when accompanied by a suboptimal response to platelet transfusion, should prompt reassessment of the underlying etiology, including immune thrombocytopenia.

The Tokyo Guidelines 2018 (TG18) provide a framework for the diagnosis, severity assessment, and management of acute cholecystitis; however, they do not specifically address the selection of source-control strategies in patients with profound thrombocytopenia.^1,2,8)^ TG18 considers early laparoscopic cholecystectomy for strictly selected patients with Grade III acute cholecystitis, including those with favorable organ system failure, low operative risk, and access to experienced surgeons at advanced centers.^1)^ Our patient was classified as having Grade III acute cholecystitis due to hematological dysfunction, which is not included in favorable organ system failure.^1,8)^ Therefore, percutaneous transhepatic gallbladder drainage followed by reassessment of the infection and platelet count could also have been considered^1,2)^; another option is endoscopic transpapillary gallbladder drainage, which avoids percutaneous puncture.^2)^ However, in this patient, transpapillary access would have been technically challenging owing to the previous distal gastrectomy with Roux-en-Y reconstruction. This procedure was not routinely performed as an emergency intervention at our institution.

Emergency open cholecystectomy was selected following platelet transfusion, as extensive gallbladder wall necrosis and pericholecystic abscess raised concerns that drainage alone might leave a necrotic infectious focus *in situ*. However, these findings did not establish that gallbladder drainage would have failed. Despite laparoscopic expertise being available, a planned open surgery was selected in view of the platelet count of 2000/μL and expected adhesions related to the previous upper abdominal surgery, to facilitate adhesiolysis, direct compression, and rapid hemostasis. Nevertheless, intraoperative blood loss reached 1570 mL, requiring transfusion of red blood cells and fresh frozen plasma. This case suggests that, when cholecystectomy is considered in patients with profound thrombocytopenia, the indication should be carefully assessed, and perioperative planning should account for the possibility of significant bleeding.

Immune thrombocytopenia is primarily a clinical diagnosis based on the medical history, physical examination, complete blood count, peripheral blood smear, and exclusion of alternative causes of thrombocytopenia.^9)^ In the present case, severe local infection and marked inflammatory response initially raised concern for infection-associated consumptive thrombocytopenia, including disseminated intravascular coagulation, and urgent source control was prioritized.^4,5,7,10)^ However, the nearly preserved coagulation times, markedly elevated fibrinogen level, absence of schistocytes, and elevated immature platelet fraction were not fully typical of advanced consumptive coagulopathy alone. Furthermore, thrombocytopenia persisted after surgical source control, and the 1-h corrected count increment of 5100 suggested a suboptimal response to platelet transfusion.^6)^ Following source control, persistent severe thrombocytopenia, a suboptimal response to platelet transfusion, and absence of platelet clumping, schistocytes, and splenomegaly all pointed to immune thrombocytopenia. Further, there were no recent changes in medications, and trilineage hematopoiesis was preserved without dysplasia or blast proliferation on bone marrow examination.^9)^ The bone marrow findings helped to exclude bone marrow failure and hematologic malignancy but were not alone diagnostic of immune thrombocytopenia. These findings emphasize that thrombocytopenia in patients with severe infection should not automatically be attributed to consumption and should be reassessed when its clinical course is inconsistent with the initial diagnosis. Corticosteroids are used as initial treatment for adult immune thrombocytopenia; rituximab is an option for an insufficient response to initial therapy.^9)^ The subsequent platelet recovery in the present case was compatible with a treatment response.

This case has some limitations. First, concomitant infection-associated platelet consumption could not be excluded. In addition, since corticosteroids, *Helicobacter pylori* eradication therapy, prednisolone, and rituximab were administered sequentially, the contribution of each treatment to platelet recovery could not be determined separately. Second, whether immune thrombocytopenia predated, was triggered by, or merely coexisted with the acute cholecystitis could not be determined. Acute and/or *Helicobacter pylori* infection may have contributed to secondary immune thrombocytopenia. Finally, a post-transfusion platelet count was not obtained before skin incision; therefore, the actual platelet count at the time of surgery was unknown. Reassessment of the platelet count after transfusion might have helped guide the choice between gallbladder drainage and immediate surgery. Furthermore, because the patient was classified as ASA-PS III, he did not meet the TG18 criteria for upfront cholecystectomy for Grade III acute cholecystitis.^1)^ The selection of emergency open cholecystectomy was based on the specific clinical circumstances of this patient and cannot be generalized to all patients with acute cholecystitis and profound thrombocytopenia. Further accumulation of similar cases is needed to clarify the appropriate source-control strategy and diagnostic approach to persistent thrombocytopenia in patients with severe infection.^3,4,6,9,10)^

## CONCLUSIONS

This case highlights the need to balance urgent source control against substantial bleeding risk in gangrenous acute cholecystitis with profound thrombocytopenia. Persistent thrombocytopenia after surgical source control, particularly with a suboptimal response to platelet transfusion, should prompt reassessment of the underlying etiology, including immune thrombocytopenia.

## DECLARATIONS

### Use of Artificial Intelligence Tools

The authors did not use generative artificial intelligence tools in the preparation of this manuscript.

### Availability of data and materials

The datasets supporting the conclusions of this article are included within the article. Further inquiries can be directed to the corresponding author.
